# Heterogeneous Changes in Mismatch Negativity Peak Latency Following Electroconvulsive Therapy: A Case Series of Three Cases

**DOI:** 10.7759/cureus.86019

**Published:** 2025-06-14

**Authors:** Yuhei Mori, Kazuko Kanno, Hiroshi Hoshino, Yuichi Takahashi, Yuhei Suzuki, Itaru Miura

**Affiliations:** 1 Department of Neuropsychiatry, Fukushima Medical University, Fukushima, JPN

**Keywords:** cognitive function, electroconvulsive therapy (ect), major depression disorder, mismatch negativity, schizophrenia

## Abstract

Mismatch negativity (MMN), a pre-attentive auditory event-related potential, is a sensitive neurophysiological marker of cortical dysfunction. Electroconvulsive therapy (ECT), which is effective for various psychiatric disorders, may transiently affect cognitive and neurophysiological processes.This case series examined MMN peak latency and cognitive outcomes following ECT in three patients with distinct psychiatric diagnoses: atypical psychosis, treatment-resistant schizophrenia, and major depressive disorder (MDD). In patients with atypical psychosis, MMN peak latency was markedly prolonged immediately after two ECT sessions and returned to baseline by 40 days, in parallel with transient cognitive decline and mild delirium. Conversely, the patient with schizophrenia demonstrated no MMN latency changes and stable cognitive scores, suggesting diminished cortical reactivity, possibly due to chronic illness or medication effects. The patient with MDD, assessed after the final two sessions of a 10-session course, exhibited sustained MMN latency prolongation with preserved cognitive function, indicating potential cumulative subclinical neural effects. These findings highlight the diagnostic and temporal heterogeneity of MMN responses to ECT, with MMN alterations not always correlating with cognitive decline. MMN may serve as a sensitive but nonspecific biomarker of neural instability during ECT, offering clinical value for the early detection of cortical vulnerability. Our results suggest that MMN monitoring, especially when interpreted alongside the diagnosis and treatment stages, may complement standard cognitive assessments and inform individualized ECT management.

## Introduction

Electroconvulsive therapy (ECT) is a well-established treatment for severe psychiatric disorders, including treatment-resistant depression and schizophrenia [1]. However, despite its efficacy, ECT is often accompanied by transient cognitive side effects, particularly during the immediate post-treatment period. These cognitive changes are typically short-lived, but can vary depending on individual vulnerabilities, treatment parameters, and diagnosis [2-5].

Mismatch negativity (MMN) is an event-related potential (ERP) that reflects pre-attentive auditory discrimination and is typically measured using the oddball paradigm [6]. It is one of the few ERPs whose neural generators and functional significance have been well established, primarily originating from the auditory cortex, including Heschl’s gyrus and the superior temporal gyrus [7,8]. Importantly, MMN is elicited automatically without requiring active participation or task comprehension, allowing its reliable measurement even in patients with cognitive impairment or disorders of consciousness. Furthermore, MMN can be recorded using relatively low-cost and non-invasive equipment, making it a practical and accessible neurophysiological marker for both research and clinical settings. MMN reflects automatic auditory deviance detection and is linked to cognitive function and clinical symptoms, particularly in schizophrenia [9-11]. It is considered to index auditory sensory memory and predictive coding mechanisms, and its impairment has been associated with deficits in attention, working memory, and executive function in various psychiatric disorders. Notably, Liu et al. found that MMN alterations after eight sessions of ECT were associated with cognitive improvement in patients with schizophrenia [12], suggesting that MMN is a potential state-dependent marker of treatment response.

Although MMN and cognitive improvements have been reported after full ECT courses [12], the immediate neurophysiological effects, when cognitive changes are subtle, remain poorly understood. Moreover, the clinical significance of early MMN alterations across diagnoses remains unclear.

We previously described a case of atypical psychosis with transient prolongation of MMN latency and temporary cognitive decline after two ECT sessions [13]. This case series extends our previous observations by evaluating the MMN and cognitive changes following two ECT sessions in patients with three distinct diagnoses. By comparing early MMN responses across different psychiatric conditions, this study aims to explore diagnostic-specific patterns and the potential utility of MMN as a sensitive biomarker for short-term neurophysiological effects of ECT. We report three diagnostically heterogeneous cases, illustrating distinct patterns of MMN change shortly after ECT, which may reflect diagnosis-specific neurocognitive responses to treatment.

## Case presentation

Patients

We enrolled three patients undergoing ECT at our institution who were diagnosed with atypical psychosis, schizophrenia, and major depressive disorder. All patients provided written informed consent for their participation and publication. This study was approved by the Ethics Committee of Fukushima Medical University.

Electroconvulsive therapy

All patients received two bitemporal ECT sessions using a Thymatron System IV device (Somatics, LLC, Venice, FL, USA). The ECT parameters were set according to standard institutional protocols. The medication regimens remained stable throughout the observation period.

MMN recording and cognitive assessment

We administered the Brief Assessment of Cognition in Schizophrenia (BACS) short form (BACS-sf) [14] and the MMN-D at baseline (the day before the first ECT treatment), the day after the two ECT treatments, and approximately one month after ECT treatment to investigate the transition of her cognitive function and the effects of ECT on MMN. The BACS-sf consists of three tests: (a) verbal memory, (b) digit sequencing, and (c) symbol coding tasks. Cognitive performance was consistently assessed using the BACS at 11:00 am, followed by an MMN recording at 1:00 pm on the same day. Additionally, clinical symptoms were evaluated using the Positive and Negative Syndrome Scale (PANSS) and Global Assessment of Functioning, except in patients with major depressive disorder, for whom the PANSS was not applicable.

Mismatch negativity

**Figure 1:**
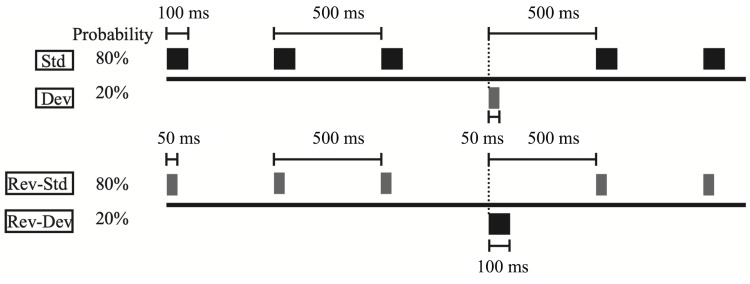
Auditory stimulus presentation scheme in the oddball paradigm for mismatch negativity recording. Overall, 4,000 standard (100 ms duration at a probability of 80%) and 1,000 deviant stimuli (50 ms duration at a probability of 20%) were presented randomly. In contrast, 1,000 reversed-standard stimuli (50 ms in duration with a probability of 80%) and 250 reversed-deviant stimuli (100 ms in duration with a probability of 20%) were presented in the same manner.

To measure mismatch negativity, auditory stimuli were presented to both ears with a constant stimulus onset asynchrony of 500 ms, using earphones (YE-103J; Nihon Kohden, Tokyo, Japan). All stimuli consisted of a sinusoidal tone at 1,000 Hz and a sound pressure level of 105 dB. Standard stimuli (100 ms duration) were presented at a probability of 80%, whereas deviant stimuli (50 ms duration) were presented at a probability of 20% (Figure 1).

Standard and deviant stimuli were presented in the same block and randomized using a computer software (multi trigger system; medical type system). Overall, 4,000 and 1,000 standard and deviant stimuli were presented, respectively. Reversed-standard stimuli (50 ms duration) and reversed-deviant stimuli (100 ms duration) were provided with probabilities of 80% and 20%, respectively (Figure 1). In total, 1,000 reversed-standard and 250 reversed-device stimuli were presented in the same manner. The patients were instructed to sit on a chair in a shielded room and concentrate on watching a self-selected silent video with lyrics during the measurements. Electroencephalography (EEG) data were recorded over 64 channels using sintered Ag/AgCl electrodes placed in a 10/10 system. The tip of the nose was used as a reference system. The sampling rate was 2,000 Hz. Electrode impedance was maintained at < 10 kΩ. EEG data were recorded with a 64-channel recorder (Neurofax EEG1218; Nihon Kohden) for offline analysis using FOCUS (Nihon Kohden). Unstable segments > 100 μV were excluded from the analysis. All data were band-pass filtered (0.5-30 Hz), referenced to the tip of the nose, segmented from 0 (onset) to 450 ms post-stimulus, and baseline-corrected to a 50 ms pre-stimulus epoch. To delineate MMN at Fz, Cz, LM (M1), and RM (M2), the ERPs elicited by the reversed-standard stimuli were subtracted from the ERPs elicited by the corresponding deviant stimuli. MMN peak latency was defined as the latency measured from the end of the shorter stimulus, that is, 50 ms after the onset of the stimulus. MMN showed the largest negative peaks at Fz and Cz in the latency range of 100-200 ms from deviant onset.

Case 1: Previously reported case, atypical psychosis

A 67-year-old woman with atypical psychosis, characterized by acute onset, good premorbid functioning, and episodic delusions with fluctuating insight, was admitted for maintenance ECT to prevent relapse [13]. Her condition did not fully meet the criteria for schizophrenia or mood disorders, and she had previously responded well to ECT. During this admission, she received two bitemporal ECT sessions (Day 3 and Day 6) using a Somatics Thymatron device (0.9 A, 0.5 ms, 30 Hz, 6.5 s, 35% dose). Adequate generalized seizures were confirmed. At baseline, her cognitive function was stable, and MMN latency was within the normal range. After two sessions, MMN latency was transiently prolonged, cognitive scores declined, and mild delirium emerged. These effects resolved within one month.

Case 2: Treatment-resistant schizophrenia

A 32-year-old man with treatment-resistant schizophrenia, characterized by persistent positive and negative symptoms and poor response to multiple antipsychotics, was admitted for maintenance ECT. He had previously experienced clozapine-induced myocarditis, leading to its discontinuation, and had shown partial symptom improvement after a full ECT course (10 sessions) in the past. During this admission, two bitemporal ECT sessions were administered on Day 4 and Day 7 using a Thymatron device (0.9 A, 0.5 ms, 30 Hz, 6.5 s, 35% dose). Generalized seizures were confirmed. His psychotropic regimen (olanzapine 10 mg, paliperidone 12 mg, clonazepam 0.5 mg) remained unchanged. MMN waveforms were poorly defined at all time points, and latency showed minimal change. BACS scores remained stable, indicating no measurable cognitive decline.

Case 3: Major depressive disorder

A 69-year-old woman with recurrent major depressive disorder and psychotic features presented with depressive mood, hypochondriacal delusions, and drug-induced oral dyskinesia. She had remained stable for over a decade after previous successful ECT, but recently relapsed following prolonged amoxapine use. Subsequent valbenazine treatment was discontinued due to drug-induced Parkinsonism, and her symptoms worsened. ECT was reinitiated due to poor response to pharmacotherapy and worsening depressive and somatic symptoms. MMN and cognitive testing were conducted during the final two ECT sessions (sessions 9 and 10). She received 10 bitemporal ECT sessions (0.9 A, 0.5 ms, 30 Hz, 6.5 s); the stimulus dose was initially 35% and increased to 40% from the fifth session. All sessions, except the fourth, elicited adequate seizures. MMN latency was markedly prolonged the day after the two sessions and remained prolonged at 40-day follow-up. BACS scores remained stable. She achieved clinical remission with no further adverse events.

**Figure 2:**
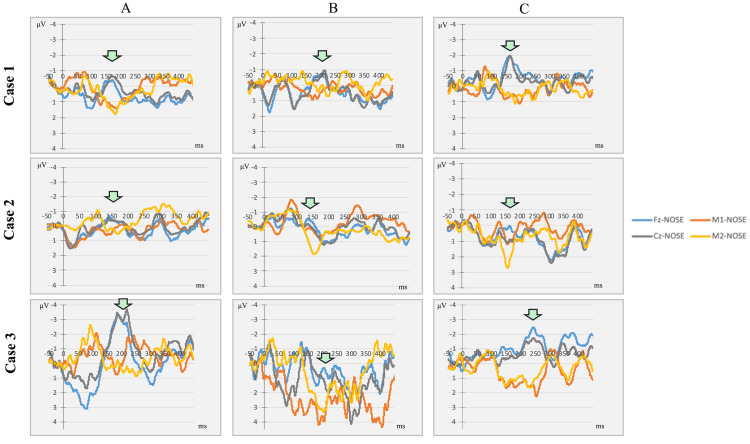
MMN difference waveforms at three time points across the three patients. These waveforms are the difference waveforms (Dev-reversed Std) obtained from the three patients in this case series. Time point A represents the baseline assessment prior to ECT, Time point B corresponds to the day after the second ECT session, and time point C reflects the follow-up evaluation conducted 40 days after the second ECT session. Arrows indicate MMN. MMN before the first ECT session (baseline) (A), on the day after the two ECT sessions (B), and 40 days after the last ECT session (C). Arrows indicate MMN peaks. In Case 1 (atypical psychosis), the MMN peak latency was transiently prolonged at B and returned to baseline at C. In Case 2 (treatment-resistant schizophrenia), the MMN waveforms were not clearly distinguishable at any time point. In Case 3 (major depressive disorder), MMN peak latency progressively increased from A to C. ECT, electroconvulsive therapy; MMN, mismatch negativity

The MMN results for each case are summarized in Figure 2.

In addition, clinical scores, MMN latency and amplitude, as well as BACS scores are presented in Table 1. 

**Table 1:** MMN, cognitive, and clinical symptom scores at three time points (baseline, after two ECT treatments, and after 40 days) across three patients. 40 Days Later refers to the assessment conducted 40 days after the second ECT session. BACS-sf, Brief Assessment of Cognition in Schizophrenia short form; ECT, Electroconvulsive Therapy; GAF, Global Assessment of Functioning; N/A, Not Applicable; MMN, Mismatch Negativity; PANSS, Positive and Negative Syndrome Scale

Measure	Time Point	Case 1 (Atypical Psychosis)	Case 2 (Schizophrenia)	Case 3 (Major Depressive Disorder)
MMN Peak Latency (ms)	Baseline	119.5	91.5	134.5
	After two ECT	159	85	172
	40 Days Later	110.5	113.5	194
MMN Amplitude (μV)	Baseline	-0.41	-0.71	-3.48
	After two ECT	-0.72	-0.57253	1.05
	40 Days Later	-1.97	0.02	-2.47
BACS – Verbal Memory	Baseline	29	32	26
	After two ECT	14	23	25
	40 Days Later	29	27	20
BACS – Digit sequencing	Baseline	17	10	12
	After two ECT	14	12	14
	40 Days Later	19	11	12
BACS – Symbol Coding	Baseline	50	32	31
	After two ECT	38	40	29
	40 Days Later	50	38	37
PANSS – Total	Baseline	35	105	N/A
	After two ECT	48	108	N/A
	40 Days Later	35	106	N/A
GAF	Baseline	85	35	70
	After two ECT	75	35	70
	40 Days Later	85	35	70

## Discussion

This case series demonstrates heterogeneous MMN responses to ECT across three diagnostic categories, providing preliminary insights into diagnosis-specific and time-dependent neurophysiological alterations. Notably, a dissociation between MMN and cognitive performance was observed in two of the three cases, highlighting the potential of MMN as a sensitive, pre-attentive marker of neural perturbation that is not necessarily mirrored by standard cognitive screening.

MMN is considered to reflect auditory sensory memory and predictive coding - functions that underlie early information processing and are often impaired in psychiatric disorders. The observed dissociation between MMN changes and cognitive test performance suggests that MMN may serve as a complementary neurophysiological biomarker, capable of capturing subtle neural disruptions even in the absence of overt cognitive decline.

In the case of atypical psychosis, MMN latency was transiently prolonged after two ECT sessions and normalized after approximately 40 days, mirroring the recovery of cognitive performance and resolution of mild delirium. This finding aligns with previous literature, suggesting that MMN can serve as an early indicator of transient cortical disruption following ECT [13].

Conversely, patients with schizophrenia exhibit minimal changes in MMN and cognitive outcomes. The MMN waveform was poorly defined at all time points, which is consistent with previous findings of attenuated MMN responses in schizophrenia, potentially due to persistent deficits in cortical plasticity or auditory deviance detection [15]. The absence of MMN or cognitive changes may reflect a floor effect, in which neurophysiological systems are already functionally compromised and less responsive to ECT.

In the case of major depressive disorder, MMN latency was persistently prolonged following the final two ECT sessions, despite stable BACS performance. This suggests that MMN may capture lingering or cumulative neurophysiological alterations not reflected in clinical cognition, particularly during the later phases of ECT. This pattern is consistent with prior reports linking MMN recovery trajectories to cognitive improvement over longer treatment courses [5,12]. The clinical threshold for meaningful MMN latency shifts has not yet been standardized [16]. However, in the present case series, latency changes ranged from minimal (<5 ms) to substantial (>30 ms), potentially reflecting diagnosis-specific differences in cortical reactivity. These findings underscore the need for future research to establish clinically relevant benchmarks for MMN latency alterations in psychiatric populations.

Collectively, these findings suggest that MMN may serve as a state-sensitive biomarker of early neural instability following ECT, particularly in diagnostically vulnerable individuals. Its dissociation from cognitive outcomes further supports its role as a complementary marker of neurophysiological integrity, rather than as a surrogate.

While this case series is limited by its small sample size (n = 3), use of brief cognitive screening tools, non-standardized treatment phases, and lack of medication control, the inclusion of diagnostically heterogeneous patients enables a comparative perspective across psychiatric categories. Furthermore, the MMN waveform in the schizophrenia patient was poorly defined, limiting its interpretability. The follow-up period was also restricted to approximately 40 days, and long-term changes remain unexplored. Moreover, potential individual-level factors, such as illness duration, cognitive reserve, or subclinical comorbidities, may have influenced the observed variability in MMN and cognitive responses. Additionally, psychotropic medications remained unchanged during the assessment period, but their long-term effects, particularly antipsychotics in the schizophrenia case and antidepressants in the MDD case, may have influenced MMN amplitude or latency. Furthermore, baseline cognitive status, treatment history, and individual cognitive reserve likely contributed to differential neurophysiological responses across cases. These potential confounding factors should be systematically controlled in future studies

We acknowledge that a larger sample - including additional diagnostic groups - would enhance the robustness and generalizability of the findings. We view this report as an exploratory step, laying the groundwork for future research incorporating expanded case series or prospective cohort designs.

## Conclusions

In summary, MMN peak latency may serve as a dynamic diagnosis-sensitive marker of cortical processing changes during ECT. Although not a surrogate for cognition, it may capture subtle neural changes before clinical symptoms emerge, offering a promising tool for individualized treatment monitoring.
